# Liquor Drinking

**Published:** 1880-09

**Authors:** 


### LIQUOR DRINKING.

IF men will drink alcohol in some shape, the least injurious time for it, is during a regular meal, or within a few minutes after, for then, the strength of the stimulus is expended on the digestive organs, and enables them to perform their work more thoroughly; hence an amount of brandy which would make one tipsy, on an empty stomach, would have no such effect if taken during dinner. But the amount taken, to be in any way beneficial, must be in proportion to the fat, butter or oils used at the same meals; in this case, it aids the system to appropriate the fat to itself, in other words, brandy taken with fatty food, tends to fatten quickly, but it does not give strength, fat people are not strong. On the other hand, it is a conceded fact in physiology, that alcohol in every shape impedes the digestion of the albuminous portion of our food, that is, brandy makes no flesh, makes no muscle, gives no strength. The prize fighter does not want fat; one main object in his training is to get rid of it and replace it with substantial muscle, with flesh, hence when in training, he never touches liquor. The advocates of brandy triumphantly point at a ruddy faced drinker with his apparently well developed muscle and well filled skin, but fat is a disease, is a puff; he has no agility of limb, no activity of body; there is no power in his arm, no courage in his heart, for he knows, and we do too, that a lean stripling or a plow boy of twenty, who was never drunk in his life "could whip him all to pieces in five minutes." Away then with all the nonsense about brandy strengthening any body, it weakens the head, it cowers the heart, and wastes away the whole man.

